# Effect of passive solar drying on food security in rural Mozambique

**DOI:** 10.1038/s41598-022-22129-9

**Published:** 2022-10-13

**Authors:** Custodio Matavel, Harald Kächele, Jonathan Steinke, Constance Rybak, Harry Hoffmann, João Salavessa, Stefan Sieber, Klaus Müller

**Affiliations:** 1 Leibniz Centre for Agricultural Landscape Research (ZALF), Eberswalder Str. 84, 15374 Müncheberg, Germany; 2 Department of Agricultural Economics, Faculty of Life Sciences, Humboldt-Universität zu Berlin, Berlin, Germany; 3 Faculty of Agrarian Sciences, Universidade Lúrio (Unilúrio), Campus Universitários de Unango Km 62, Sanga District, Niassa, Mozambique; 4 Eberswalde University for Sustainable Development, Eberswalde, Germany; 5 Faculty of Health Sciences, Universidade Lúrio (Unilúrio), Nampula, Mozambique

**Keywords:** Energy and society, Solar thermal energy, Devices for energy harvesting, Sustainability

## Abstract

Achieving food security in Mozambique is critical, since 80% of the population cannot afford an adequate diet. While increasing agricultural production is a necessary effort to address this challenge, inadequate post-harvest treatment leads to storage losses and quality degradation, with repercussions for food security. The use of solar drying is promoted as a solution to provide efficient and reliable access to food preservation that improves the food security situation in rural communities. However, there is a lack of clear evidence on how the use or access to solar drying affects food security. This study identifies the determinants of farmers’ choice to use solar drying and evaluates the effect of a passive solar dryer on food security using survey data from 634 households. We allocated solar dryers to selected communities and all interested individuals belonging to these communities were eligible to use it. Propensity score matching and endogenous switching poisson regression are used to estimate the average effect. The use of solar drying with associated training significantly increases the food security status of participants by increasing household food availability, women’s dietary diversity, and months of adequate household food provision and by decreasing the household food insecurity access scale.

## Introduction

Achieving food security (FS) is a complex development challenge in Mozambique^1^. According to World Food Programme, 80% of people in Mozambique cannot afford an adequate diet—defined as a diet that is rich in diverse foods necessary to meet the nutritional needs of an individual^2,3^—and 42.3% of children under the age of 5 are stunted^4^. Despite efforts to increase agricultural production^5^, high levels of post-harvest losses (PHL) in Mozambique limit the poor population’s access to quality food, thereby exacerbating food insecurity^6^. For example, PHL of maize result from traditional storage practices, where insects, rodents, and mold infection can destroy up to 60% of the harvest^7^.

Increased productivity could potentially mitigate PHL, but this remains challenging for many farmers, who face labor constraints and limited access to modern agricultural inputs^8^. While agriculture in Mozambique has ample scope to increase its productivity, efforts to increase farm output are thus unlikely to suffice for achieving long term FS in rural areas. Especially considering the need for ecological efficiency, reducing PHL seems a key feature of an integrated FS strategy. Increasing rural residents’ access to locally viable postharvest processing and preservation solutions may therefore be a sustainable strategy to improve FS^9^.

The introduction of appropriate technologies to provide efficient and reliable access to food preservation is particularly critical for rural populations, but current standard technologies require access to electricity or fossil fuels^10^. Solutions that require electricity are more efficient in theory, but they are not applicable in rural Mozambique, where 67% of the population lives^11^, but only 6% of the population has access to electricity^12^. Appropriate technologies are those that match the needs of users with the available resources, within a specific context^13^. Thus, in many remote locations, such as in rural Mozambique, it is crucial to provide farmers access to locally adapted agro-processing technologies^9^ as well as affordable and sustainable energy solutions for agricultural and domestic uses^14^, in order to achieve the main dimensions of FS.

Scalable and affordable solutions that do not require electricity or fossil fuels for food processing are already available. The use of solar energy for drying agricultural produce has been proposed as a cost-effective and environmentally sustainable solution to increase shelf-life, minimize food and specific nutrient losses and health risks, as well as add value to agricultural products^15–17^. As opposed to refrigeration, which requires a continuous supply of energy, it is attractive since after initial drying, no further equipment or energy input is required to maintain product quality^18^. Nevertheless, the widely practiced method of drying agricultural products in rural Mozambique is the open-sun drying (OSD)^19^. This method has significant limitations since it can lead to high product losses due to inadequate drying, fungal growth, as well as the encroachment of insects, birds, and rodents^20^.

Several types of devices that use solar energy to dry food products, the so called passive solar dryers (PSD), have already been developed and tested, with their superiority over the often-used OSD proven. Solar drying, for example, results in a product with comparably better quality^21^, takes less time to finalize the drying process^22^, reduces the dependence on weather conditions^15^, prevents harm to the product from external factors such as rain, wind, dust, and insects^23^, and reduces contamination by toxins^24^, among other advantages. In light of the broad evidence on the technical performance of PSD, development organizations and governments already promote PSD to contribute to improvements in FS^25^. Empirical evidence on the impacts of these initiatives on the target group’s FS status is scarce, however. Nagwekar et al*.*^26^ demonstrate that the use of solar drying can significantly increase dietary diversity during the lean season due to extended preservation period of diverse food, but the study did not investigate effects on other aspects of FS. To date, the general lack of evidence limits the capacity of policy-makers and development practitioners concerned with rural FS to make informed decisions on investments and interventions. To be able to prioritize locally suitable FS solutions, decision-makers need evidence on the heterogeneous impacts of alternative intervention options, including PSD. To fill this gap, this study takes an experimental approach to analyze the effects of PSD on four indicators of FS, reflecting the four dimensions of FS in rural Mozambique. By providing an integrated analysis of FS effects across the four pillars, this study goes beyond existing impact studies of PSD. To inform future efforts toward effective introduction and scaling of PSD approaches by development organizations, this study also aims to identify factors that influence rural households’ decisions to use a PSD. With this, our analysis provides decision-makers the evidence needed for deciding on whether to implement PSD interventions, and how to maximize adoption.

## Results

### Food security status of PSD users and non-users

The household FS categories, classified via Food Availability Scores (FAS), Women Dietary Diversity Score (WDDS), Months of Adequate Household Food Provisioning (MAHFP), and Household Food Insecurity Access Scale (HFIAS), are presented in Table 1. The majority of solar dryer users (56%) has a high availability of food, whereas most non-users have low availability of food. Only 5% of non-users fall into the high availability category. As for MAHFP, the majority of users are moderately food insecure (58%), while in the most food insecure category the majority are non-users. Nevertheless, we observed only a small difference between the users and non-users in the percentage of least food insecure households. The classification based on HFIAS revealed that 21% of users and 53% of users fall within food secure and mildly food secure categories, respectively. The majority of non-users (70%) are severely food insecure. As for the WDDS, medium to high diversity is observed among women belonging to households that used the solar dryer, whereas the majority of women (71%) in the group of non-users are classified into low dietary diversity. A Chi-square test provides evidence of statistically significant differences between users and non-users in all FS indicators (P < 0.01).

**Table 1:** Prevalence of food security.

FS indicator	Row labels	Non-users (%)	Users (%)
FAS	High availability	5	56
	Low availability	95	44
MAHFP	Least food insecure	6	2
	Moderately food insecure	29	58
	Most food insecure	66	40
HFIAS score	Food secure	1	21
	Mildly food insecure	8	53
	Moderately food insecure	21	22
	Severely food insecure	70	4
WDDS	High dietary diversity	7	59
	Medium dietary diversity	22	41
	Lowest dietary diversity	71	0

### Determinants of households’ choice to use the solar dryer

The results of probit regression present the predicted likelihood of ever using solar drying (Table 2). These results indicate that households headed by female or older farmers, with a larger family size, are more likely to use PSD. The size of agricultural land and the percentage of agricultural sales are also positively related to the use of PSD. Farmers who had training during project implementation or who had information from neighbors and those who belong to an association or cooperative are also more likely to use the solar dryer. Production of staple foods also influences adoption. The production of maize, beans, and rice increases the probability of adopting solar dryers.

**Table 2:** Determinants of predicted likelihood of using solar dryer (probit model output).

Variable	Coef.	Std. err.
Geographic location (Lioma = 1)	− 0.23	0.16
Gender of household head (male = 1)	− 0.28**	0.15
Age of household head (years)	0.04***	0.00
% of agricultural output sold	1.82***	0.36
Size of household	0.08***	0.03
Size of land (ha)	0.29***	0.05
Received training from project (yes = 1)	0.28**	0.13
Use drying methods (yes = 1)	0.15	0.13
Received information from neighbor (yes = 1)	0.36***	0.13
Received info from extension (yes = 1)	− 0.03	0.13
Belong to an association/cooperative (yes = 1)	0.39***	0.14
Produce beans (yes = 1)	0.62***	0.14
Produce maize (yes = 1)	− 0.43***	0.14
Produce sorghum (yes = 1)	0.03	0.14
Produce rice (yes = 1)	0.29**	0.13
Produce cassava (yes = 1)	− 0.05	0.14
_cons	− 4.27***	0.42

***Significant at 1%; **Significant at 5%; *Significant at 10%. The actual P value are presented at Supplementary Data S1.

### The effect of using PSD on FS

**Figure 1:**
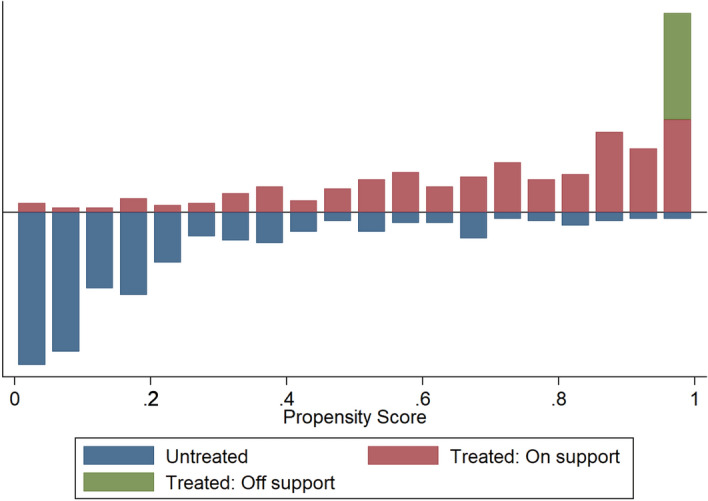
Distribution of estimated propensity scores and region of common support.

Matching of PSD users and non-users was undertaken within a region of common support in order to ensure that treated and control households are comparable in their covariates that predict use of solar dryer (see Supplementary Data S1). The test of assumptions indicate that the balancing property of the propensity score is satisfied. The distribution of propensity scores and the region of common support is presented in Fig. 1. A substantial overlap of the propensity scores of both groups can be observed.

The difference in FS status between the self-selected solar dryer users and non-users is estimated using nearest neighbor matching, radius matching, and kernel-based matching. The Average Treatment Effect on Treated (ATT) estimates are presented in Table 3. The use of solar dryer has a significant positive effect on FAS and WDDS as well as a significant negative effect on HFIAS. The ATT for FAS is 9.57 in the nearest neighbor matching, 14.02 in the radius matching, and 9.38 in the kernel-based matching. Furthermore, positive and statistically significant ATT is found for WDDS in all the matching methods. The ATT in the nearest neighbor matching is 1.98, in the radius matching 2.74, and in the kernel-based matching 1.96. This results imply that the use of solar dryer improves household food availability and women’s dietary diversity. Furthermore, on average, the HFIAS score of treated households is 6.15 points lower than the HFIAS score of matched control households in the nearest neighbor matching. In the radius and kernel-based matching, the ATT is − 6.51 and − 6.54, respectively. Nevertheless, according to nearest neighbor and kernel matching, the use of the solar dryer was not sufficient to ensure that all household members had three or more meals a day during the 12 months prior to the survey, as measured by MAHFP. In radius matching, the use of the solar dryer has a positive and significant effect of 1.66.

**Table 3:** Effect of using solar dryer on food security based on PSM.

FS indicator	Nearest neighbor matching		Radius matching		Kernel-based matching	
	ATT	Std. err.	ATT	Std. err.	ATT	Std. err.
FAS	9.57***	1.15	14.02***	0.23	9.38***	0.71
MAHFP	0.43	0.53	1.66***	0.14	0.53	0.44
HFIAS score	− 6.15***	0.76	− 6.51***	0.20	− 6.54***	0.64
WDDS	1.98***	0.276	2.74***	0.07	1.96***	0.19

***Significant at 1%; **Significant at 5%; *Significant at 10%. The actual P value are presented at Supplementary Data S1.

The use of PSM does not account for unobservable factors. Thus, we estimated ATT from the endogenous-switching regression (ESR), which accounts for both observed and unobserved factors. Based on the Wald test, the null hypothesis of no correlation between the treatment errors and the outcome errors could not be rejected for HFIAS and MAHFP, implying that there was no presence of selection bias arising from unobserved factors. However, for FAS and WDDS, the Wald test is significant at 5% and 1%, respectively, indicating possible biases from unobserved factors (see Supplementary Data S2 for detailed output results). The ATTs estimated from ESR are presented in Table 4.

**Table 4:** Effect of using solar dryer on food security based on ESR.

FS indicator	ATT	Std. err.
FAS	14.31***	0.46
MAHFP	2.3***	0.65
HFIAS score	− 5.47**	2.14
WDDS	2.61***	0.12

***Significant at 1%; **Significant at 5%; *Significant at 10%. The actual P value are presented at Supplementary Data S2.

The ATT for FAS, MAHFP, and WDDS are positive and statistically significant at 1%, whereas the ATT for HFIAS score is negative and statistically significant at 5%. These results are similar to those found in radius matching, despite small differences in the ATT. In general, however, the use of the solar dryer positively affected the FS status, which can be seen from the negative ATT for HFIAS score and the positive ATT for FAS, WDDS, and MAHFP.

## Discussion

This study identifies the factors that influence rural households’ decisions to use a passive solar dryer and investigates the effect of using a passive solar dryer on food security. It was conducted in a drought-prone area^27^, where food provision is dependent on rainfed agriculture. This area has an improved FS during the harvest season^28^, thus, we focus our analysis on the period of greatest food shortage (immediately prior to the harvest).

The results suggest that household characteristics, such as age, gender, size of household, size of land, market orientation, training, neighbor-to-neighbor communication, membership in an association, and the production of staple foods increase the likelihood of adopting solar drying. These findings are consistent with previous research on the determinants of agricultural technology adoption^29–33^.

Older farmers are more experienced and, therefore, may have greater awareness of the benefits of new technologies, as well as more resources to enable adoption^33^. Moreover, in the context of this study, one solar dryer per community may not have been enough for every interested farmer and the most powerful members of the community—i.e. the older farmers—may have been granted the right of first access. Likewise, farm size might be an indicator of the level of economic resources owned by households^34^, thus, farmers with larger farms may also be more likely to adopt new technologies^35^. Other studies also show a positive impact of farm size on agricultural technology adoption^36,37^. Contrary to what is found in previous studies^38,39^, female headed households are more likely to use the new technology than men. This is consistent with the results in Table 3, in which 40% of non-users and 65% of users were female. However, to capture the actual gender effect, the dynamics of the decision-making process need further examination by, for example, accounting for the number male or female adults within the households^39^. Household size in terms of the number of household members, may be an indicator of family labor availability^40^. In addition, some household members may be engage in off-farm activities that provide additional income^34^. As more resource-endowed households have a better ability to cope with production and price risks, they are more willing to adopt new technologies^41^.

The importance of cooperatives in promoting adoption is already demonstrated^30,31^. Post-harvest technologies require more skills in operation and management, thus demanding greater organizational competence. Hence, members of a cooperative can benefit from systematic and frequent training as well as regular collaborative actions^30^. Farmers who sell their products are probably more interested in adopting post-harvest technologies as these could help them to maintain the quality of their products and, consequently, their competitiveness in the market. Moreover, better storage quality also means that farmers do not have to sell right immediately after harvest (when prices are low) but they can keep their product and sell it at a later time, when prices are high.

Neighbors can be the most influential source of information when choosing to adopt an agricultural technology. Early technology adopters provide a community laboratory from which neighbors can gain some experience^42^. Farmers without the required skills to operate a new technology may face some difficulties to use it^43^. Thus, farmers who got training on solar drying technology are more willing to adopt.

Staple foods are the biggest source of food security in the region, thus encouraging farmers who produce these foods to become interested in adopting technologies that allow them to reduce PHL and maintain the quality of their products as long as possible. Therefore, the probability of adopting the solar dryer is higher for households producing staple foods. Nonetheless, maize farmers were less likely to use the PSD than others, probably because it is a very popular and important crop, with households already having significant experience with alternative post-harvest management to reduce losses. Thus, they may not have much interest in adopting a new technology.

The use of the solar dryer has, on one hand, the potential to alleviate seasonal food shortages and increase food shelf life, hence enhancing food availability and stability. On the other hand, it adds value to agricultural products, thus increasing marketability and providing better financial returns for farmers^9,44^. Household financial returns are an important determinant of food access^45^. Moreover, solar drying results in products with improved nutrient quality and hygiene^46^. PSM is employed to estimate the average treatment effect of using a solar dryer on FS. This technique assumes that there is no selection bias arising from unobservable factors. The results suggest that using a solar dryer is positively related to FS. Specifically, there is strong evidence (significant at the 1% level) of a positive FS effect, measured by FAS, HFIAS, and WD. However, evidence is mixed for MAHFP. The nearest neighbor and kernel-based matching did not show a statistically significant effect of solar drying on MAHFP, whereas in the radius matching, the effect was positive and significant. It is not uncommon for different matching techniques to result in estimates of different magnitudes. Except for MAHFP, the PSM results are internally coherent and in line with our expectations. The type of PSM technique substantially affects the magnitude of treatment effect but none of the methods is a priori superior to the others^47^. Thus, this study also uses ESR to complement PSM and check the robustness of the estimates. ESR accounts for both unobservable and observable factors, providing ATTs that are closer to radius matching. This might imply that nearest neighbor and kernel-based matching underestimate the average effect of solar drying on FS. In general, however, both methods also show that the use of a solar dryer positive affects FS. This might be because the study participants who chose to use the solar dryer could protect themselves from the unavailability and inaccessibility of food in the lean months thereby increasing their FAS and WDDS and decreasing HFIAS score as compared to non-users. This complies with results of Nagwekar et al*.*^26^, who find that the use of solar drying increases WDDS by 36%. Although the impact of solar drying on MAHFP is not significant in the nearest neighbor and kernel-based matching, the radius matching and ESR estimates suggest that solar drying can provide some FS stability by guaranteeing that food is available over a 12 months period. It should be noted that the 12 months recall period also includes some months before the intervention for those farmers who decided to use the solar dryer several months after the allocation. Moreover, only a small difference (4%) is observed between users and non-users with regards to the percentage of least food secure households (Table 4). This could explain the non-significance in the nearest neighbor and kernel-based matching. Nevertheless, the use of PSD did not show a clear effects on the stability dimension, although the HFIAS score also include some elements of stability since it also analyses respondents' perceptions of household food insecurity experiences over a 30-day period^48^.

One of the limitations of the solar dryer implemented in the study area is that it requires several rounds of drying to dry large quantities of food. In addition, since it requires initial investment cost, the dryer was shared among households, which may not have allowed them to dry enough product for the entire lean period. This may discourage new users from joining and continuing the use of the solar dryer. In fact, two of the main limitations of PSD are the small amount they dry per drying process and the intermittent nature of solar radiation availability^49^. Hence, there is a need to develop solar dryers with relatively high efficiency that dry relatively large quantities of product in the shortest time possible in order to minimize seasonal food insecurity, such as the natural convection solar tunnel dryer and the passive glass-roof type greenhouse dryer^50^. Moreover, the additional use of adequate storage and packaging materials can improve moisture removal and thermal efficiency and prevent eventual food loss during storage^51,52^. Further monitoring of food quality after the drying process is necessary. Studies on the longevity and life of the dryer are also necessary, since this can impact the feasibility and sustainability of such a technologies. FS can also affect the financial returns for the farmers^9,44^. Thus, we also suggest that future research examines the impact of solar drying on household income. The use of the FS outcome, such as the anthropometric measurements, may also provide more evidence on the impact of using solar drying on FS.

## Conclusion

This study demonstrates the potential of passive solar dryers to contribute to improved food security in rural Mozambique. Robust evidence for positive effects on all four pillars of food security suggests that promoting passive solar dryers, along with trainings on their use, can be a promising intervention strategy for improving the wellbeing of relatively food-insecure subsistence farmers. This finding underscores that food security and nutrition outcomes can be improved with simple, locally available technology and without the need to intensify local production or food imports, which may have negative environmental side-effects. To benefit large numbers of rural households, however, massive introduction of passive solar dryers, possibly in larger scale, would be needed. Future research could evaluate the costs of such an intervention, as well as the longevity and resilience of the drying devices over extended use periods, to assess its cost–benefit ratio against alternative food security interventions. With adequate support from government authorities or humanitarian organizations, the construction and diffusion of passive solar dryers could possibly become an income generation model for rural youth and landless population.

## Methods

### Study area

This study was conducted in Gurué district, which is located at around 15° South and 36° East, in Zambézia Province, Central Mozambique between December 2021 and January 2022. First, Zambézia Province was selected for this study due to its high levels of chronic malnutrition^53^ and its frequent food shortages^54^; thus, the need for interventions to improve the FS situation. Secondly, Gurué district was selected because it generates a large surplus during harvest season^55,56^, but faces a high risk losing it due to the commonly used OSD method^57^. Thirdly, two of the three administrative posts in Gurué district, Lioma and Mepuagiua, are also purposively selected due to their characteristics in terms of low urbanization rates and high proportions of the population engaged in agricultural activities. According to data provided by the local authorities, Lioma is divided into 29 communities and comprises 29,868 inhabitants, while Mepuagiua is divided into 11 communities with 61,227 inhabitants. In this region, about 90% of the population practices small-scale agriculture as the main occupation^28^ and few households have access to electricity^58^. The average farm size is less than 2 ha and, generally, food insecure households predominate during the pre-harvest period; during the harvest season there is a predominance of households with medium and high food security^28^. Local diets usually consists of maize and cassava, both cooked as a paste and served with beans or dark green leaves sauces and/or dried or fresh fish^27^.

### Study design and sampling approach

In this study, we allocate a solar dryer to 50% of communities in each administrative post (15 communities in Lioma and 6 in Mepuagiua). The communities were randomly selected and solar dryers were built and allocated to local leaders in August 2020. Allocation to communities (here defined as clusters) was made since allocation to individuals was not financially feasible^59^. The solar dryer used, hereby wooden and locally produced dryers that allow an indirect drying of food, is already tested and approved by local residents in the study area. This solar dryer was intended to be used during harvest periods, so from implementation to the last data collection two harvest periods were observed. Therefore, individuals who had utilized the dryers in at least one of the two harvest periods were considered as users. A detailed description of the solar dryer is presented in previous work^22^.

All interested individuals belonging to the selected communities were eligible to use the solar dryer. Likewise, they could choose whether they wanted to use it, not use it, leave or refuse to participate in the study at any time. As such, in each community, assignment into the treatment group (users of the solar dryer) was based on farmers’ self-selection. Since farmers had no previous experience with the implemented solar dryer design, as this was the first implementation, the choice to use was voluntary and gradual. Therefore, we only conducted a survey after intervention, from December 2021 to January 2022. This period coincides with the period when the highest levels of food insecurity are observed^57^. A total of 367 households had used solar dryers since their construction between August and September 2020, of which 308 (84%) agreed to be part of the study: 155 households were from Mepuagiua and 153 were from Lioma. Similarly, 350 of the households that did not use the solar dryer were randomly selected into the control group (non-users), of which 326 (93%) agreed to be part of the study. Thus, the total number of observations is 634. The survey captured household socio-economic characteristics, demographics, and four FS indicators to capture the multidimensionality of FS. It was performed in accordance with the guidelines laid down in the ‘Declaration of Helsinki’ and ethically reviewed by the Mozambican National Committee of Bioethics in Health (IRB00002657, Ref 370/CNBS/19). Informed consent was obtained from all individuals who agreed to participate in the study.

### Conceptual framework

**Figure 2:**
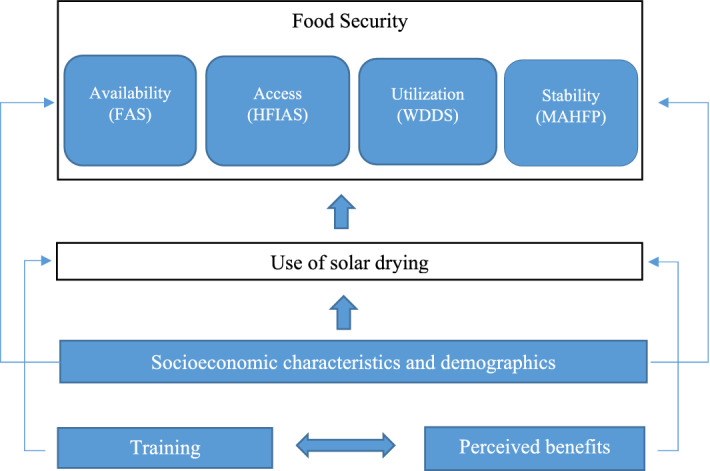
Conceptual framework for causality between solar dryer use and food security.

Figure 2 presents a conceptual background summarizing the hypothesized causal effects of using a solar dryer on FS. A number of socioeconomic and demographic factors can influence farmer’s decision to use a solar dryer. Likewise, individual perceptions of the benefits of using a solar dryer and training can also influence the choice to use it. Socioeconomic and demographic factors together with the use of the solar dryer can determine the physical availability of food, its physical access, economic access, and intra-household food allocation to individuals. These food security dimensions were captured using Food Availability Scores (FAS), Household Food Insecurity Access Scale (HFIAS), and Women Dietary Diversity Score (WDDS). The use of a solar dryer can allowed users to keep a higher share of their harvest at home instead of having to sell it in fear of losing it to pests and mold, thus contributing to stability across time. This temporal dimension of FS was captured via Months of Adequate Household Food Provisioning (MAHFP). Detailed description of each FS indicator is found in the following subsections.

#### Food availability scores (FAS)

To assess food availability, we used a Household Food Inventory (HFI), which is based on participant self-reporting of any food present in the house at time of the survey^60,61^. A predefined list (Table 5), containing 46 foods items generally available and consumed in the study area, was used. This list was generated following a baseline data collection in February 2020. Respondents were asked whether the foods on the list were present in their home or not and what was the source (purchased, borrowed, own production or donation/offered) of each respective item. In case of any doubt, the respondents could consult someone they considered most likely to have the answer or check the storage facilities or any other place where they were likely to have the food. The FAS ranged from 0 to 46 and was calculated as the total sum of foods available at the households. The household was classified according to their food availability as low (if FAS < 24) or high (if FAS ≥ 24), similar to Gichunge et al*.*^62^ and Koui et al*.*^63^.

**Table 5:** Household food inventory.

No.	Food group	Food items
1	Starchy staples	Cassava, bread, corn flour, maize, noodles, rice, white potatoes, sorghum
2	Dark green leafy vegetables,	Amaranth, cassava leaves, cabbage, lettuce, pumpkin leaves, sweet potato leaves
3	Other vitamin A rich fruits and vegetable	Carrots, sweet potatoes
4	Other fruits and vegetables,	Garlic, tomato, onion, banana, eggplant, okra
5	Eggs	Eggs
6	Organ meat	Liver, kidney, heart or other organ meats
7	Meat and fish	Beef (cow), chicken, duck, pigeon, fish, goat, sheep/lamb, pork
8	Legumes, nuts and seeds	Beans, cowpeas, green grams, pigeon peas, soybeans, groundnuts, coconuts, sunflower, bambara nuts
9	Milk and milk products	Milk, yogurt

#### Household Food Insecurity Access Scale (HFIAS)

The access dimension of FS was assessed via Household Food Insecurity Access Scale (HFIAS). This is a nine-item food insecurity scale that uses a recall period of four weeks to answer both occurrence and frequency questions^64^. The respondents were first asked whether the condition in the question occurred at all in the past four weeks and, if the answer was “yes”, they were subsequently asked to answer whether the condition happened rarely (once or twice), sometimes (three to ten times), or often (more than ten times) in the past four weeks. The detailed questionnaire is presented in Supplementary Table S1.

To ensure that the respondent understood the meaning of interview questions, key informants (extension officers and community leaders) were asked to review and adapt the phrases and definitions to local context. To further refine the questions and gain insights into whether the questions were actually being understood as intended, 10 individuals who are not part of the study sample were asked to answer the questionnaire in order to allow the interviewers to explore the respondent’s own understanding of the question and its meaning, according to the Key Informant Interview Guide^64^. HFIAS scores, ranging from 0 to 21, were calculated for each household by summing the codes for each frequency-of-occurrence question. The household were categorized into Food Secure, Mildly Food Insecure, Moderately Food Insecure, and Severely Food Insecure, according to the Household Food Insecurity Access Scale Indicator Guide^64^.

#### Women dietary diversity score (WDDS)

Assessment of individual dietary intakes within households allows for a more accurate estimation of intra-household food utilization^65^. Thus, in this study we use individual dietary diversity scores for women to capture food utilization. These are good proxies for overall dietary quality in a poor rural context and are linked to nutritional status^66^.

One female respondent aged 15 years or older was interviewed within selected households. When there was more than one eligible and available woman in the household, the respondent was randomly selected. The households that were visited each day during the survey were also randomly selected to minimize day to day differences. We asked questions about all foods they consumed the previous day, inside and outside the home. The food were grouped into 9 groups (see Table 1) and, consequently, respondents were assigned numbers ranging from 0 to 9 according to the number of food groups they consumed. Women who consumed less than 4 food groups were classified into low dietary diversity, those who consumed 4 to 5 food groups as medium dietary diversity, and more than 6 as high dietary diversity^67^. Appropriate wording of the questions was agreed with local leaders and extension officers, with the food groups listing locally available foods and locally recognized names for each.

#### Months of adequate household food provisioning (MAHFP)

Months of adequate household food provisioning (MAHFP) was used to capture the stability of food consumption over time. Respondents were asked to identify in which of the last 12 months all household members could have at least three meals a day. This allowed us to assess food stability and classify households into most food insecure (if MAHFP ≤ 5), moderately food insecure (6 ≤ MAHFP ≤ 9), or least food insecure (10 ≤ MAHFP ≤ 12), as described in previous work^28^.

### Determinants of households’ choice to use the solar dryer

A farmer is expected to use the solar dryer if the benefits or utility gain of using it outweighs that of not using it. This utility, in terms of improving food safety, can be expressed as a function the observable vector of covariates (Z):

1 $$SDU_{i}^{*} = \alpha Z_{i} + \mu_{i} ,\quad SDU_{i} = 1\;[SDU_{i}^{*} > 0]$$

$SDU_{i}$ is a binary variable representing use of the solar dryer by household $i$. $\alpha$ is a vector of parameters to be estimated, $Z_{i}$ is a vector variables that are expected to influence the use of solar drying, and $\mu_{i}$ is the error term assumed to be normally distributed (see Tables 6 and 7). These variables are selected as they are shown in previous studies to potentially influence adoption of agricultural technologies (e.g. Zhang et al*.*^30^, Launio et al*.*^68^, Karki et al*.*^69^, Pollard et al*.*^70^, Gitonga et al*.*^71^ and Hamza Conteh et al*.*^72^).

**Table 6:** Descriptive statistics of surveyed households (continuous/count variables).

Variable	Non-users (n = 335)				PSD users (n = 308)			
	Mean	Std. dev.	Min	Max	Mean	Std. dev.	Min	Max
Age of household head (years)	34.46	13.55	18	83	52.72	16.56	18	82
% of agricultural output sold	0.59	0.19	0	0.96	0.69	0.19	0.09	0.99
Size of household	6.01	2.42	1	13	6.94	2.60	1	15
Size of land (ha)	1.45	1.12	0.2	7	2.77	1.76	0.25	7

**Table 7:** Descriptive statistics of surveyed households (dummy variables).

Variable	% of Non-users	% of Users
Lioma residents	47	50
Female household heads	40	65
Received training from project	44	59
OSD users	44	50
Received information from neighbor or relatives	49	64
Received information from extension	50	52
Belong to an association/cooperative	19	43
Beans farmers	16	49
Maize farmers	74	54
Sorghum farmers	48	50
Rice farmers	29	52
Cassava farmers	45	52

### Estimation of effects of solar dryers on FS

To determine the FS effects of PSD, we were interested in identifying what level of FS would the PSD users have in its absence by comparing the FS situation between PSD users and non-users. Since the decision to use the solar dryer is based on farmers’ own perceptions about the effectiveness and benefits of this decision, the random allocation of households to the users group was not possible. In such case, self-selectivity issues need to be addressed to minimize biases on the impact estimations^73^. Propensity score matching (PSM), along with generalized propensity score and instrumental variable, are econometric approaches that address selection bias in cross-sectional data^74^. Generalized propensity score is applied in the case of continuous treatment^75^. To implement a valid instrumental variable analysis, it is necessary to find a reliable instrument, which is complicated and not always possible^76^. Therefore, in this study, we apply the PSM approach^77^ to assess the impact of using a solar dryer on FS. It is recommended in situations where self-selection bias is an issue, since it compares the difference between the outcome variables of users and non-users with similar inherent and observable characteristics^78^. However, one issue related to the use of PSM is that unmeasured or unobserved characteristics are likely correlated with both the treatment and outcome variables, which is likely to introduce bias, thus understating or overstating the program’s effect^79^. Thus, to complement the propensity score matching and check the robustness of the results, an endogenous switching regression (ESR) model is also employed.

#### Propensity score matching (PSM)

We first generated the propensity score using a probit model. Second, the average treatment effect on the treated (ATT), based on the predicted propensity scores, was estimated. PSM is suitable in situation where the baseline data is not available^80^. To check the robustness of the results, we use three different matching algorithms, namely nearest neighbor matching, radius matching, and kernel-based matching. PSM is expressed as:

2 $${\text{p}}\;\left( {{\text{X}}_{{\text{i}}} } \right) = \Pr \;\left( {SDU = 1{|}X} \right) = E\;(SD|X)$$

where $SDU$ is a dummy variable indicating the use solar dryer. $X$ represents the factors that are expected to influence the adoption of solar dryer (see Table 1). Two central conditions of the PSM is the assumption that observations with the same propensity score must have the same distribution of observable characteristics independently of treatment status (Eq. 3) and assignment to treatment is unconfounded (Eq. 4).

3 $$SDU \bot X| p\left( X \right)$$

4 $$Y_{1} ,\;Y_{0} \bot SD| X$$

The user written program package *PSMATCH2* in STATA^81^ was used to estimate the propensity score.

For impact evaluation, it is desirable to estimate the average impact of solar dryer adoption on PSD users, the so called average effect of the treatment on the treated (ATT)^82^, which can be estimated as follows:

5 $$ATT = E\left\{ {Y_{1i} - Y_{0i} {|}SDU_{i} = 1} \right\} = E\left[ {E\left\{ {Y_{1i} - Y_{0i} {|}SDU_{i} = 1, \;p\left( {X_{i} } \right)} \right\}} \right] = E\left[ {E\left\{ {Y_{1i} {|}SDU_{i} = 1, \;p\left( {X_{i} } \right)} \right\} - E\left\{ {Y_{0i} {|}SDU_{i} = 0, \; p\left( {X_{i} } \right)} \right\}|SDU_{i} = 1} \right]$$

where $\tau$ is the ATT, with $Y_{1i}$ and $Y_{0i}$ being the potential outcomes (FAS, HFIAS score, MAHFP, and WDDS) in the two counterfactual situations of using solar dryer and not using, respectively. The ATT was estimated for each outcome variable separately.

#### Endogenous switching regression (ESR) model

The ESR model considers selectivity as an omitted variable problem^83^. Thus, it was used to capture the differential response taking into accounts unobserved variables that can influence both treatment and outcome variables. In this study, all the outcomes variables are the food security indicators resulting from the count of positive responses to the questionnaire questions (see Sect. 5.3). Thus, an Endogenous Switching Poisson Regression (ESPR) approach was adopted. The ATT was estimated using the STATA command *etpoisson,* which estimates the coefficient of an endogenous binary treatment model when the outcome is a count variable^84,85^. The estimated model can be stated as follows:

6 $$E\left( {Y_{j} {|}X_{ij} ,\;SDU_{j} , \in_{j} } \right) = exp\left( {X_{ij} \beta_{i} + \alpha SDU_{j} + \in_{j} } \right)$$

where $Y_{j}$ denotes the food security status, $X_{ij}$ represents the vector of the independent variables, and $\in_{j}$ is the error term.

## Supplementary Information

Supplementary Information 1.

Supplementary Information 2.

Supplementary Information 3.

Supplementary Table S1.

## Data Availability

All data generated or analyzed during this study are included in this published article (and its Supplementary Information files).
