# Supplementary material for: Effect of passive solar drying on food security in rural Mozambique
Source: Sci Rep. 2022 Oct 13;12:17154. doi: 10.1038/s41598-022-22129-9 (PMC9561633; doi:10.1038/s41598-022-22129-9)
Supplement: Supplementary file 4 — Supplementary Table S1. [file 41598_2022_22129_MOESM4_ESM.docx]

Supplementary Table1: Household Food Insecurity Access Scale (HFIAS) Measurement Tool

| No | Question | Response Options | Code |
| --- | --- | --- | --- |
| 1. | In the past four weeks, did you worry that your household would not have enough food? | 0 = No (skip to Q2)  1=Yes | ....\|___\| |
| 1.a | How often did this happen? | 1 = Rarely (once or twice in the past four weeks)  2 = Sometimes (three to ten times in the past four weeks)  3 = Often (more than ten times in the past four weeks) | ....\|___\| |
| 2. | In the past four weeks, were you or any household member not able to eat the kinds of foods you preferred because of a lack of resources? | 0 = No (skip to Q3)  1=Yes | ....\|___\| |
| 2.a | How often did this happen? | 1 = Rarely (once or twice in the past four weeks)  2 = Sometimes (three to ten times in the past four weeks)  3 = Often (more than ten times in the past four weeks) | ....\|___\| |
| 3. | In the past four weeks, did you or any household member have to eat a limited variety of foods due to a lack of resources? | 0 = No (skip to Q4)  1=Yes | ....\|___\| |
| 3.a | How often did this happen? | 1 = Rarely (once or twice in the past four weeks)  2 = Sometimes (three to ten times in the past four weeks)  3 = Often (more than ten times in the past four weeks) | ....\|___\| |
| 4. | In the past four weeks, did you or any household member have to eat some foods that you really did not want to eat because of a lack of resources to obtain other types of food? | 0 = No (skip to Q5)  1 = Yes | ....\|___\| |
| 4.a | How often did this happen? | 1 = Rarely (once or twice in the past four weeks)  2 = Sometimes (three to ten times in the past four weeks)  3 = Often (more than ten times in the past four weeks) | ....\|___\| |
| 5. | In the past four weeks, did you or any household member have to eat a smaller meal than you felt you needed because there was not enough food? | 0 = No (skip to Q6)  1=Yes | ....\|___\| |
| 5.a | How often did this happen? | 1 = Rarely (once or twice in the past four weeks)  2 = Sometimes (three to ten times in the past four weeks)  3 = Often (more than ten times in the past four weeks) | ....\|___\| |
| 6. | In the past four weeks, did you or any other household  member have to eat fewer meals in a day because there was not enough food? | 0 = No (skip to Q7)  1=Yes | ....\|___\| |
| 6.a | How often did this happen? | 1 = Rarely (once or twice in the past four weeks)  2 = Sometimes (three to ten times in the past four weeks)  3 = Often (more than ten times in the past four weeks) | ....\|___\| |
| 7. | In the past four weeks, was there ever no food to eat of any kind in your household because of lack of resources to get food? | 0 = No (skip to Q8)  1=Yes | ....\|___\| |
| 7.a | How often did this happen? | 1 = Rarely (once or twice in the past four weeks)  2 = Sometimes (three to ten times in the past four weeks)  3 = Often (more than ten times in the past four weeks) | ....\|___\| |
| 8. | In the past four weeks, did you or any household member go to sleep at night hungry because there was not enough food? | 0 = No (skip to Q9)  1=Yes | ....\|___\| |
| 8.a | How often did this happen? | 1 = Rarely (once or twice in the past four weeks)  2 = Sometimes (three to ten times in the past four weeks)  3 = Often (more than ten times in the past four weeks) | ....\|___\| |
| 9. | In the past four weeks, did you or any household member go a whole day and night without eating anything because there was not enough food? | 0 = No (questionnaire is finished)  1=Yes | ....\|___\| |
| 9.a | How often did this happen? | 1 = Rarely (once or twice in the past four weeks)  2 = Sometimes (three to ten times in the past four weeks)  3 = Often (more than ten times in the past four weeks) | ....\|___\| |
